# Early nasogastric tube feeding in optimising treatment for hyperemesis gravidarum: the MOTHER randomised controlled trial (Maternal and Offspring outcomes after Treatment of HyperEmesis by Refeeding)

**DOI:** 10.1186/s12884-016-0815-1

**Published:** 2016-01-27

**Authors:** Iris J. Grooten, Ben W. Mol, Joris A. M. van der Post, Carrie Ris-Stalpers, Marjolein Kok, Joke M. J. Bais, Caroline J. Bax, Johannes J. Duvekot, Henk A. Bremer, Martina M. Porath, Wieteke M. Heidema, Kitty W. M. Bloemenkamp, Hubertina C. J. Scheepers, Maureen T. M. Franssen, Martijn A. Oudijk, Tessa J. Roseboom, Rebecca C. Painter

**Affiliations:** Department of Obstetrics and Gynaecology, Academic Medical Centre, University of Amsterdam, Amsterdam, The Netherlands; Department of Clinical Epidemiology, Biostatistics and Bioinformatics, Academic Medical Centre, University of Amsterdam, Amsterdam, The Netherlands; The Robinson Institute, School of Paediatrics and Reproductive Health, University of Adelaide, Adelaide, Australia; Laboratory of Reproductive Biology, Academic Medical Centre, University of Amsterdam, Amsterdam, The Netherlands; Department of Obstetrics and Gynaecology, Medical Centre Alkmaar, Alkmaar, The Netherlands; Department of Obstetrics and Gynaecology, VU Medical Centre, VU University Amsterdam, Amsterdam, The Netherlands; Department of Obstetrics and Gynaecology, Erasmus MC, Erasmus Medical Centre Rotterdam, Rotterdam, The Netherlands; Department of Obstetrics and Gynaecology, Reinier de Graaf Hospital, Delft, The Netherlands; Department of Obstetrics and Gynaecology, Máxima Medical Centre, Veldhoven, The Netherlands; Department of Obstetrics and Gynaecology, Radboud University Medical Centre, Nijmegen, The Netherlands; Department of Obstetrics and Gynaecology, Leiden University Medical Centre, Leiden, The Netherlands; Department of Obstetrics and Gynaecology, Maastricht University Medical Centre, Maastricht, The Netherlands; Department of Obstetrics and Gynaecology, University Medical Centre Groningen, Groningen, The Netherlands

**Keywords:** Hyperemesis, Nausea and vomiting in pregnancy, Tube feeding, Intravenous rehydration, Effectiveness, Outcomes

## Abstract

**Background:**

Hyperemesis gravidarum (HG), or intractable vomiting during pregnancy, is the single most frequent cause of hospital admission in early pregnancy. HG has a major impact on maternal quality of life and has repeatedly been associated with poor pregnancy outcome such as low birth weight. Currently, women with HG are admitted to hospital for intravenous fluid replacement, without receiving specific nutritional attention. Nasogastric tube feeding is sometimes used as last resort treatment. At present no randomised trials on dietary or rehydration interventions have been performed. Small observational studies indicate that enteral tube feeding may have the ability to effectively treat dehydration and malnutrition and alleviate nausea and vomiting symptoms. We aim to evaluate the effectiveness of early enteral tube feeding in addition to standard care on nausea and vomiting symptoms and pregnancy outcomes in HG patients.

**Methods/Design:**

The MOTHER trial is a multicentre open label randomised controlled trial (www.studies-obsgyn.nl/mother). Women ≥ 18 years hospitalised for HG between 5 + 0 and 19 + 6 weeks gestation are eligible for participation. After informed consent participants are randomly allocated to standard care with intravenous rehydration or early enteral tube feeding in addition to standard care. All women keep a weekly diary to record symptoms and dietary intake until 20 weeks gestation. The primary outcome will be neonatal birth weight. Secondary outcomes will be the 24-h Pregnancy Unique Quantification of Emesis and nausea score (PUQE-24), maternal weight gain, dietary intake, duration of hospital stay, number of readmissions, quality of life and side-effects. Also gestational age at birth, placental weight, umbilical cord plasma lipid concentration and neonatal morbidity will be evaluated. Analysis will be according to the intention to treat principle.

**Discussion:**

With this trial we aim to clarify whether early enteral tube feeding is more effective in treating HG than intravenous rehydration alone and improves pregnancy outcome.

**Trial registration:**

Trial registration number: NTR4197. Date of registration: October 2^nd^ 2013.

**Electronic supplementary material:**

The online version of this article (doi:10.1186/s12884-016-0815-1) contains supplementary material, which is available to authorized users.

## Background

Nausea and vomiting in pregnancy (NVP) is common, affecting 50–80 % of pregnancies [1]. Often these symptoms are mild and self-limiting and resolve without intervention in the second trimester. In other cases however, severe intractable vomiting can lead to dehydration, electrolyte disturbances and significant weight loss necessitating hospital admission. The condition of intractable vomiting during pregnancy is called hyperemesis gravidarum (HG) [2]. HG has repeatedly been associated with poor pregnancy outcome including low birth weight (LBW, <2500 g: OR 1.42), small for gestational age (OR 1.28) and prematurity (OR 1.32) [2–4]. Furthermore, HG has a major impact on maternal wellbeing and quality of life [5–7] and remains the largest single cause of hospital admission in early pregnancy [8, 9]. However, the aetiology of HG is poorly understood [10–12].

Approximately 0.8–2 % of all pregnancies are complicated by HG [2]. Currently, there are no treatments with proven efficacy available according to the latest Cochrane review on interventions for nausea and vomiting in early pregnancy [1]. Hospitalisation can be required for intravenous treatment of dehydration and electrolyte imbalance. Currently, women who suffer from HG do not receive any particular nutritional attention, although enteral tube feeding is sometimes used as a treatment of last resort [2, 13]. Enteral tube feeding effectively treats both dehydration and malnutrition in non-pregnant patients with poor intake [14] and has been shown to be safer than parenteral nutrition in pregnancy [15]. Moreover, in several small studies in women with HG, which did not employ a control group, it alleviated symptoms and was well tolerated if continued in a home setting [16–18]. There have been no controlled trials to investigate the extent to which enteral tube feeding can positively affect pregnancy outcome and maternal quality of life, nausea and vomiting symptoms or time in hospital.

At present, there is no evidence on the effectiveness and efficiency of rehydration and dietary interventions for HG. We hypothesise that enteral tube feeding in addition to standard care is a more effective treatment for HG symptoms than standard care with intravenous rehydration alone, and improves pregnancy outcome.

This multicentre randomised controlled trial (RCT) aims to compare early enteral tube feeding in addition to standard care, with standard care alone. Outcomes of interest are birth weight and maternal nausea and vomiting symptoms, maternal quality of life, duration of hospitalisation, weight gain and neonatal morbidity. The study is conducted within the Dutch Consortium for Studies in Obstetrics, Fertility and Gynaecology (www.studies-obsgyn.nl).

## Methods/Design

### Participants/ eligibility criteria

Patients ≥ 18 years of age are eligible if they have been admitted to hospital because of HG (first admission or readmission) at a gestational age between 5 + 0 and 19 + 6 weeks. Patients with singleton or multiple pregnancies are eligible. A diagnosis of HG is made if excessive nausea or vomiting necessitates hospital admission, in the absence of any other obvious cause such as drug induced vomiting or infection.

Exclusion criteria are mola hydatidosa pregnancy, non-vital pregnancy, acute infection causing vomiting (e.g. appendicitis, pyelonephritis), contraindication for enteral tube feeding (e.g. oesophageal varices, allergies to enteral tube mix compounds) or HIV infection.

### Procedures, recruitment and randomisation

**Fig. 1:**
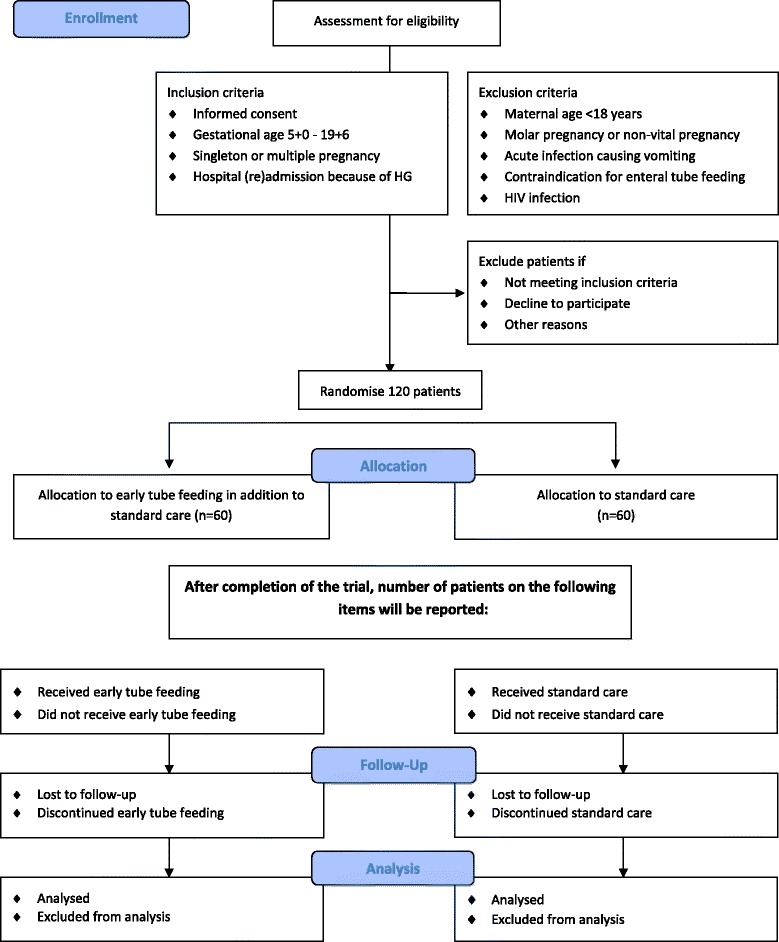
CONSORT 2010 flow diagram MOTHER trial

This study is a nationwide multicentre open label RCT conducted within the Dutch Consortium for Studies in Obstetrics, Fertility and Gynaecology, a nationwide collaboration of hospitals in the Netherlands. The staff and/or local research coordinator of the participating hospitals identifies eligible women. After counselling and reading the patient information form, patients are asked for written informed consent. Patient information is provided in Dutch and English. See Fig. 1.

Randomisation is performed by a web based computerised program using permuted-block randomisation. Randomisation is allocated in a 1:1 ratio for standard care or enteral tube feeding in addition to standard care, with a block size of four. Stratification according to centre is applied.

### Intervention

Participants are allocated to standard care or enteral tube feeding in addition to standard care. Standard care consists of intravenous rehydration and, when considered necessary, laboratory monitoring, electrolyte and/or vitamin supplementation, antiemetic medication and dietetic advice. Type of rehydration regimen, medication and duration of hospitalisation is prescribed according to local protocol. In case of prolonged hospital admission or readmissions, tube feeding can be initiated at the decision of the attending physician.

When allocated to the intervention group, participants receive a nasogastric tube as soon as possible after randomisation, in addition to standard care. If the initial nasogastric tube is dislocated or poorly tolerated a nasoduodenal or nasojejunal insertion can also be considered. Tube feeding regimen and mix is prescribed according to local protocol. As soon as tube feeding is tolerated and participants have received safety instructions (e.g. recognising symptoms that need to be evaluated in hospital, because of potential tube blockage, dislocation or aspiration), discharge home with tube feeding is encouraged under the guidance of a hospital dietician. Energy intake per tube is continued at least until the patient is able to maintain an oral intake of 1000 cal per day for one week. According to the NICE guideline on nutrition, tube feeding in a home setting is considered to be safe [14].

### Data collection

At the day of randomisation, participants fill out a questionnaire. This questionnaire consists of validated NVP symptom and NVP specific quality of life measures (24-h Pregnancy Unique Quantification of Emesis and nausea score, PUQE-24; Hyperemesis Impact of Symptoms questionnaire, HIS; Nausea and Vomiting in Pregnancy Quality of Life questionnaire, NVPQoL) [7, 19–21], psychopathology (Hospital Anxiety and Depression Scale, HADS; Symptoms CheckList 90, SCL-90) [22–25] and general health related questions (Short Form 36, SF-36; EuroQol 5 Dimensions questionnaire,EQ5D) [26, 27].

Participants fill out additional questionnaires (NVPQoL, HIS, HADS) 1 and 3 weeks after randomisation and record a diary at weekly intervals (PUQE-24, weight, medication use, dietary intake) from randomisation until 20 weeks gestation. If dietary intake has normalised from 15 weeks gestation onwards, this is no longer recorded. Six weeks postpartum (HADS, SF-36, EQ5D) and 12 months postpartum a final questionnaire is filled out (HADS, SF-36, EQ5D, SCL-90). See Table 1.

**Table 1:** Time line MOTHER trial

	T0	T1	T2	T3	T4	T5	T6	T7
	*Randomisation*	*+1 week*	*+ 2 weeks*	*+ 3 weeks*	*+ 4 weeks until GA 20 weeks*	*Birth*	*6 weeks post-partum*	*12 months post-partum*
Diary								
PUQE	X	X	X	X	X			
Current weight	X	X	X	X	X			
Medication use	X	X	X	X	X			
Dietary intake	X	X	X	X	X^a^			
Questionnaires								
General health	X							
NVPQoL	X	X		X				
HIS	X	X		X				
HADS	X	X		X			X	X
SF-36	X						X	X
EQ5D		X					X	X
SCL-90	X							X
Biobank material								
Maternal blood	X							
Cord blood						X		
Placental biopsies						X		

*PUQE* pregnancy unique quantification of emesis and nausea score, *NVPQoL* nausea and vomiting in pregnancy quality of life questionnaire, *HIS* hyperemesis impact of symptoms questionnaire, *HADS* hospital anxiety and depression scale, *SF-36* short form 36, *EQ5D* euroQol 5 dimensions questionnaire, *SCL-90* symptoms checklist 90

^a^If dietary intake has normalised from GA 15 weeks onwards, this will be no longer recorded

To evaluate potential HG and birth weight predictors, detailed information on obstetric and medical history, anthropometrics (before and during pregnancy), antiemetic medication use, given treatment(s) (including intravenous and/or tube feeding regimen and tube location), laboratory results, treatment and pregnancy complications and birth outcomes are collected using a standardised Case Report Form (CRF; see Additional file 1). Research staff obtains the information needed based on medical and dietician records. Maternal demographics (ethnicity, education level, marital status), mode of conception and onset of nausea and vomiting symptoms are enquired via the questionnaire.

All participants in this trial are asked for informed consent of storage of maternal blood (taken with routine laboratory analysis during hospital admission for HG), cord blood and placental biopsies (taken at birth) in an obstetrical biobank (the Preeclampsia and Non-preeclampsia Database, Academic Medical Centre Amsterdam, the Netherlands). The addition of these samples will enable molecular studies in HG aetiology and consequences. Furthermore, cord blood will be used for the assessment of plasma lipids (cholesterol, HDL, LDL, triglycerides, Apolipoprotein A and B), glucose, leptin and thyroid function (TSH, fT4).

## Outcome measures

### Primary outcome measure

The primary outcome will be neonatal birth weight.

### Secondary outcome measures

Secondary outcomes will be the validated PUQE-24 score one week after randomisation, maternal weight gain, dietary intake, HIS, NVPQoL, EQ5D, SF-36, HADS and SCL-90 scores, urinary ketones, duration of hospital stay and number of readmissions. Furthermore, gestational age at birth, preterm birth rate, small for gestational age (SGA; <10^th^ percentile) placental weight, umbilical cord plasma lipids, neonatal hypoglycaemia, hyperbilirubinaemia and congenital anomalies will be evaluated. Lastly, we will evaluate maternal side effects of tube feeding and intravenous rehydration and reasons for discontinuation of the allocated treatment.

### Follow-up of infants

A plan for long-term follow up of children is in preparation, because little is known about the long term health effects of babies born to mothers whose pregnancies were complicated by HG and we have reason to hypothesise that maternal malnutrition during early pregnancy has long term effects on the offspring’s cardiometabolic health [4]. Funding for follow-up has not yet been obtained.

## Statistical issues

### Sample size

The sample size is based on a difference in mean birth weight of 200 g (SD 400 g) between the intervention group and the control group, which we consider clinically relevant. With a beta of 0.2 and alpha of 0.05 and a possible 10 % loss to follow up, we need to randomise 120 participants (60 per arm). This sample size is also large enough to detect a two point reduction in PUQE-24 score 1 week after randomisation (maximum 15 points, SD 3 points) and differences in quality of life, psychopathology and general health questionnaires ≥ 10 %.

### Data analysis

Data will be analysed according to the intention to treat principle. Difference in birth weight will be assessed using parametric testing. PUQE-24 score will be analysed using multivariate regression and repeated measurements ANOVA or mixed models, as will be quality of life assessments. Other secondary outcomes will be addressed in a similar manner. For non-normally distributed variables non-parametric equivalents will be used. To evaluate the potential of each of the strategies, we will also perform a per protocol analysis, taking into account only those women that were treated according to protocol.

### Data safety monitoring committee

Serious Adverse Events (SAEs) are reported to the Data Safety Monitoring Committee (DSMC). The DSMC can decide, if indicated, to terminate the trial prematurely.

### Ethical considerations

This trial has been approved by the ethics committee of the Academic Medical Centre Amsterdam (Reference number MEC AMC 2012_320) and by the boards of management of all participating hospitals. The trial is registered in the Dutch Trial Register, NTR4197, http://www.trialregister.nl*webcite*. Date of registration: October 2^nd^ 2013. The full protocol can also be downloaded from the study website: www.studies-obsgyn.nl/mother.

## Discussion

Since HG is the largest cause of hospital admission in early pregnancy and has major consequences for maternal quality of life, with possible adverse effects on birth outcomes, evidence based treatment options are needed. Optimal treatment should be safe, reduce maternal complaints, duration of hospital stay and minimise adverse effects on offspring health. This trial will provide evidence on these subjects comparing standard care with intravenous rehydration and early enteral tube feeding in addition to standard care.

## Additional files

Additional file 1:
**Case Report Form MOTHER trial.** (PDF 252 kb) Additional file 2:
**CONSORT 2010 checklist MOTHER trial.** (DOCX 48 kb)

## Abbreviations

CRF: case report form
EQ5D: euroQol 5 dimensions questionnaire
HADS: hospital anxiety and depression scale
HG: hyperemesis gravidarum
HIS: hyperemesis impact of symptoms questionnaire
NICE: National Institute for Health and Care Excellence
NVP: nausea and vomiting in pregnancy
NVPQoL: nausea and vomiting in pregnancy quality of life questionnaire
PUQE-24: 24-h pregnancy unique quantification of emesis and nausea score
SCL-90: symptoms checklist 90
SF-36: short form 36
