# Supplementary material for: Early nasogastric tube feeding in optimising treatment for hyperemesis gravidarum: the MOTHER randomised controlled trial (Maternal and Offspring outcomes after Treatment of HyperEmesis by Refeeding)
Source: BMC Pregnancy Childbirth. 2016 Jan 27;16:22. doi: 10.1186/s12884-016-0815-1 (PMC4730616; doi:10.1186/s12884-016-0815-1)
Supplement: Additional file 1: — Case Report Form MOTHER trial. (PDF 252 kb) [file 12884_2016_815_MOESM1_ESM.pdf]

Case Number |__|__| __| - |__| __| __|

Case Report Form

CONFIDENTIAL

Mother

Maternal and Offspring outcomes after Treatment of HyperEmesis by Refeeding

Principal investigator:
Prof. dr. T.J. Roseboom

Coordinating researcher:
I J Grooten, MD PhD student

Contact

I J Grooten, MD PhD student
Tel: 020-5668483 / 06-22971381
Email: mother@studies-obsgyn.nl
Website: www.studies-obsgyn.nl/mother

Please read CRF instructions carefully!

Verwijderd: 1
Verwijderd: 17
Verwijderd: 04

TABLE OF CONTENTS

TABLE OF CONTENTS 2
CRF INSTRUCTIONS 3
1. General information 4
2. Randomization 4
3. Medical history 5
4. Obstetric history 5
5. Current pregnancy 6
5.1 Baseline characteristics 6
5.2 Urinary ketones at admission (during which patient was randomized) 7
5.3 Blood test at admission (during which patient was randomized) 7
5.4 Medication during first admission (during which patient was randomized) 8
6. Intervention (started during admission in which patient was randomized) 9
6.1 Intravenous (i.v.) drip 9
6.2 Tube feeding 11
7. Level of care 13
7.1 Routine pregnancy check-ups 14
7.2 Hospital readmissions for HG 14
8. Delivery 16
9. Neonatal birth data 18
10. Hospital admission postpartum 19
10.1 Maternal hospital admission 19
10.2 Neonatal hospital admission 20
11. (Serious) Adverse Events 21
12. End Study Form 22
12.1 Study stop 22
13. Supplement for patients included before 01-09-2014 23
13.1 Maternal background 23
13.2 Current pregnancy 24

Verwijderd: 1
Verwijderd: 17
Verwijderd: 04

CRF INSTRUCTIONS

1. Fill out CRF using a blue or black pen
2. Write clearly, if possible using capitals
3. Answer all questions. Only use 'unknown' when this option is given
4. selection of buttons should be: ☑ or ☒ or ⊗(if applicable)
5. Radio buttons 'O' are used when only <u>one</u> answer is possible
6. Check boxes '☐' are used when <u>multiple</u> answers are possible
7. Open Combs, e.g. '|__|' are used for numerical answers
8. For missing numeric values, write '-1'. Otherwise, select 'unknown'
9. Write dates as follows: dd-mm-jj e.g. 01-01-2001
10. When a date is (partly) missing, use '99' for missing part, e.g. when the day is missing: 99-01-2001. When day and month are missing: 99-99-2001. When complete date is missing: 99-99-99
11. Never hide corrections (using Tipp-Ex)
12. When you want to correct something, strike through false answer with a single line, followed by the right answer. Sign and date this correction
13. After CRF completion, the local PI has to sign the CRF to declare complete and truthful filling out. After signing, corrections can no longer be made to the CRF

Verwijderd: 1

Verwijderd: 17

Verwijderd: 04

1. General information

Case number |__|__|__| - |__|__|__| *(clinic - case)*

Date of birth |__|__| - |__|__| - |__|__| *(dd-mm-yy)*

Patient initials |__|__| *(initials first name and female family name)*

2. Randomization

Date of randomization |__|__| - |__|__| - |__|__| *(dd-mm-yy)*

Treatment allocation
O Tube feeding + standard care
O Standard care

Randomized on day of hospital admission for hyperemesis gravidarum (HG)*
O no O yes O unknown
|
Date of hospital admission for HG |__|__| - |__|__| - |__|__| *(dd-mm-yy)*

Date of hospital discharge for HG |__|__| - |__|__| - |__|__| *(dd-mm-yy)*

Current admission is first admission for HG
O no O yes O unknown
|
Number of previous admissions for HG |__|__| *(0-10)*

*Hyperemesis gravidarum will further be abbreviated as HG

Verwijderd: 1
Verwijderd: 17
Verwijderd: 04

3. Medical history

Disease(s)*

Hypothyroidism	O no	O yes
Hyperthyroidism	O no	O yes
Pre-existing diabetes (type I/type II)	O no	O yes
Pre-existing hypertension	O no	O yes
Peptic ulcer	O no	O yes
Depressive disorder†	O no	O yes
Anxiety disorder§	O no	O yes
Eating disorder	O no	O yes

Other; ______________________________________

*History of disease or ongoing disease
†Including postnatal depression
§Including posttraumatic stress disorder (PTSS)

4. Obstetric history

**Gravidity**	|__|__| *(0-15)*
**Parity**	|__|__| *(0-10)*
**Miscarriage**	|__|__| *(0-10)*
**EUG**	|__|__| *(0-10)*
**Termination of pregnancy** *(APLA)*	|__|__| *(0-10)*
**Progeniture** *(children alive)*	|__|__| *(0-10)*

Previous pregnancy
O no O yes
|
HG in a previous pregnancy
O no O yes O unknown
|
Requiring hospital admission
O no O yes O unknown

Verwijderd: 1
Verwijderd: 17
Verwijderd: 04

5. Current pregnancy

5.1 Baseline characteristics

Estimated date of delivery* |__|__| - |__|__| - |__|__| *(dd-mm-yy)*
**Based on ultrasound or embryo transfer*

Type of pregnancy
O Singleton
O Twins / Higher order multiple

Height* |__|__|__| cm *(140-210)*
**unknown: -1*

Smoking
O No/Quit before pregnancy O Yes O Unknown

Drug use
O No/Quit before pregnancy O Yes O Unknown
↓
☐ Cannabis
☐ Other

Folic acid use
O No O Yes O Unknown

Antiemetics started before admission *(medicatie tegen misselijkheid)*
O No O Yes O Unknown

Verwijderd: 1
Verwijderd: 17
Verwijderd: 04

5.2 Urinary ketones at admission (during which patient was randomized)

Date first measurement of urinary ketones during admission process*†

|__|__| - |__|__| - |__|__| *(dd-mm-yy)*
** Unknown: 99-99-99*
†May or may not be the same as admission date

Fill out corresponding value for urinary ketones

O Negative O Positive O Unknown
|
O +
O ++
O +++
O ++++
O Unknown

5.3 Blood testing at admission (during which patient was randomized)

Date first blood test during admission process*†

|__|__| - |__|__| - |__|__| *(dd-mm-yy)*
** Unknown: 99-99-99*
†May or may not be the same as admission date

Fill out corresponding blood values for*

Hb	|__|__|.|__|	*mmol/l*
Ht	|__|.|__|__|	*l/l*
TSH	|__|.|__|__|	*mU/l*
Na	|__|__|__|	*mmol/l*
K	|__|.|__|	*mmol/l*
ASAT	|__|__|__|	*U/l*
ALAT	|__|__|__|	*U/l*
Urea	|__|.|__|	*mmol/l*
Creatinin	|__|__|__|	*mmol/l*

**Unknown: -1*

Have magnesium (Mg) and phosphate (P) been measured during admission

O No O Yes O Unknown
|
Date of first measurement*† |__|__| - |__|__| - |__|__| *(dd-mm-yy)*
** Unknown: 99-99-99*
†May or may not be the same as first blood test date. In some hospitals, Mg and P are only tested in patients receiving tube feeding

Fill out corresponding blood values for*

Mg *(magnesium)*	|__|.|__|	*mmol/l*
P *(fosfaat/fosfor)*	|__|.|__|	*mmol/l*

**Unknown: -1*

Verwijderd: 1
Verwijderd: 17
Verwijderd: 04

5.4 Medication during first admission (during which patient was randomized)

Antiemetics *(medicatie tegen misselijkheid)*

O No O Yes O Unknown

|

☐ Emesafene p.o. / supp
☐ Primperan p.o. / supp
☐ Primperan i.v.
☐ Ondansetron (Zofran) p.o. / supp
☐ Ondansetron (Zofran) i.v.
☐ Steroids*
☐ Other

**Prescribed as antiemetic, not because of fetal lung ripening*

Vitamins

O No O Yes O Unknown

|

☐ B1 *(thiamin)*
☐ B6 *(pyridoxin)*
☐ B12 *(cobalamin)*
☐ B complex
☐ Other

Verwijderd: 1
Verwijderd: 17
Verwijderd: 04

6. Intervention (started during admission in which patient was randomized)

6.1 Intravenous (i.v.) drip

Note: For exact intravenous rehydration regimen, check both medical and nursing record to help you fill out this section

Did patient receive an i.v. drip*

O No O Yes O Unknown
|
O Patient declined
O Doctor advised against
O Other; ______________________

**As standard treatment or in combination with tube feeding*

If i.v. drip:

Was i.v. drip placed on day of randomization

O No O Yes O Unknown
|
Date of i.v. drip placement* |__|__| - |__|__| - |__|__| *(dd-mm-yy)*
**Unknown: 99-99-99*

i.v. drip solution contained*

☐ NaCl
☐ Glucose
☐ KCl
☐ Other; ______________________

**If in medical or nursing record only: 'i.v. drip according to protocol', fill out what is prescribed according to local protocol for HG*

Did patient experience any side effect(s) of i.v. drip

O No O Yes* O Unknown
|
☐ Phlebitis (pain/redness/swelling at insertion area)
☐ Allergic reaction
☐ Other; _______________

** See paragraph 8.3 whether SAE form needs to be filled out*

Verwijderd: 1
Verwijderd: 17
Verwijderd: 04

Was i.v. drip removed because of side effects

O No O Yes O Unknown

|

O Patients request
O Doctors' advice
O Other; _______________

Date of i.v. drip removal*† |__|__| - |__|__| - |__|__| *(dd-mm-yy)*

**Unknown: 99-99-99*

†All reasons for i.v. drip removal

If i.v. drip was removed, was it replaced

O No O Yes O Unknown

|

Date of i.v. drip replacement*† |__|__| - |__|__| - |__|__| *(dd-mm-yy)*

**Unknown: 99-99-99*

†Replacement during this admission, <u>not</u> placement of i.v. drip during readmission. In some hospitals patients receive a venflon and return the next day again for rehydration. This is not considered i.v. drip replacement but continuation of care until venflon is removed

Date of replaced i.v. drip removal*† |__|__| - |__|__| - |__|__| *(dd-mm-yy)*

**Unknown: 99-99-99*

†All reasons for i.v. drip removal

Verwijderd: 1
Verwijderd: 17
Verwijderd: 04

6.2 Tube feeding

> ***Note:** The dietician in your hospital will fill out a weekly registration form for all patients in this trial that have received tube feeding. When you fill out a CRF, please collect these form(s) for the specific patient, to help you fill out this section. These forms also contain complementing information on tube feeding regimen and caloric intake (not asked in this CRF)*
>
> ***Please attach the dietician registration form(s) to this CRF***

If randomized for standard care: Did patient receive a tube

O No O Yes O Unknown

|

O Patients request
O Doctors'/dieticians' advice
O Other; ________________________

If randomized for tube feeding + standard care: Did patient receive a tube

O No O Yes O Unknown

|

O Patient refused tube placement
O Tube not in stock
O Other; ________________________

If tube:

Was tube placed on day of randomization

O No O Yes O Unknown

|

Date of tube placement* |__|__| - |__|__| - |__|__| *(dd-mm-yy)*

**Unknown: 99-99-99*

Did patient experience any side effect(s) of tube

O No O Yes* O Unknown

|

☐ Nose/throat irritation
☐ Continuation of vomiting
☐ Tube obstruction
☐ Tube dislocation
☐ Aspiration
☐ Intestinal bleeding
☐ Intestinal perforation
☐ Other; ________________

** See paragraph 8.3 whether SAE form needs to be filled out*

Verwijderd: 1
Verwijderd: 17
Verwijderd: 04

If tube was dislocated, how often did this occur* |__|__| *times (0-10)*
** Unknown: -1*

Was tube removed because of side effects
O No O Yes O Unknown
|
O Patients request
O Doctors' advice
O Other; _______________

Date of tube removal*†§ |__|__| - |__|__| - |__|__| *(dd-mm-yy)*
**Unknown: 99-99-99*
†All reasons for tube removal
§Patients are normally discharged with tube in situ. Date may be later than date of discharge

If the tube was removed, was it replaced
O No O Yes O Unknown
|
Date of tube replacement*† |__|__| - |__|__| - |__|__| *(dd-mm-yy)*
**Unknown: 99-99-99*
†Tube replacement directly following tube removal (within 48 hours), not tube placement during readmission

Date of replaced tube removal*†§ |__|__| - |__|__| - |__|__| *(dd-mm-yy)*
**Unknown: 99-99-99*
†All reasons for tube removal
§Patients are normally discharged with tube in situ. Date may be later than date of discharge.

Was a duodenal or jejunal tube placed at any point*
O No O Yes O Unknown
|
O At initial tube placement (instead of nasogastric tube)
O At replacement

**In general a nasogastric tube is placed*

Reason(s) for duodenal or jejunal tube placement
☐ Nasogastric tube dislocation
☐ Continuation of vomiting
☐ Other; _______________

Verwijderd: 1
Verwijderd: 17
Verwijderd: 04

7. Level of care

Did patient return/go to primary care after remission (or stabilization) of HG <u>during the course of pregnancy</u>

O No O Yes O Unknown

|

Date of return to primary care* |__|__| - |__|__| - |__|__| *(dd-mm-yy)*

**Unknown: 99-99-99*

Was patient <u>afterwards</u> referred to secondary/tertiary care

O No O Yes O Unknown

|

Date of referral* |__|__| - |__|__| - |__|__| *(dd-mm-yy)*

**Unknown: 99-99-99*

Was there a medical indication(s) for (continuation of) antenatal/perinatal care <u>after 20 weeks gestation</u> in a secondary/tertiary center

O No* O Yes O Unknown

|

☐ Continuation of HG
☐ PIH
☐ PE/HELLP
☐ IUGR
☐ Diabetes (gravidarum/type I/type II)
☐ Premature rupture of membranes
☐ Threatening preterm labour
☐ Placenta previa (marginalis/totalis)
☐ Vaginal bleeding $2^{nd}/3^{th}$ trimester
☐ Placental abruption (partial/total)
☐ Breech/transverse position
☐ Other

** No secondary/tertiary care, or patients wish for secondary/tertiary care without medical indication*

Verwijderd: 1
Verwijderd: 17
Verwijderd: 04

7.1 Routine pregnancy check-ups

Was maternal weight measured during <u>routine pregnancy check-ups</u> (primary/secondary/tertiary care)

O No O Yes O Unknown

|

**Measurement**	**Weight *** *kg (35-250)* **Unknown: -1*	**Date of measurement*** *(dd-mm-yy)* **Unknown: -1*
1	|__|__|__|	|__|__|-|__|__|-|__|__|
2	|__|__|__|	|__|__|-|__|__|-|__|__|
3	|__|__|__|	|__|__|-|__|__|-|__|__|
4	|__|__|__|	|__|__|-|__|__|-|__|__|
5	|__|__|__|	|__|__|-|__|__|-|__|__|
6	|__|__|__|	|__|__|-|__|__|-|__|__|
7	|__|__|__|	|__|__|-|__|__|-|__|__|
8	|__|__|__|	|__|__|-|__|__|-|__|__|

7.2 Hospital readmissions for HG

Number of readmissions for HG* |__|__| *(0-10)*

**After admission in which patient was randomized*

Fill out dates of readmission(s) because of HG

**Readmission***	**Hospital admission** *(dd-mm-yy)*	**Discharge home** *(dd-mm-yy)*
1	|__|__|-|__|__|-|__|__|	|__|__|-|__|__|-|__|__|
2	|__|__|-|__|__|-|__|__|	|__|__|-|__|__|-|__|__|
3	|__|__|-|__|__|-|__|__|	|__|__|-|__|__|-|__|__|
4	|__|__|-|__|__|-|__|__|	|__|__|-|__|__|-|__|__|
5	|__|__|-|__|__|-|__|__|	|__|__|-|__|__|-|__|__|

**If more than 5 readmissions, please print/copy this page another time to fill out dates of subsequent readmissions*

Verwijderd: 1
Verwijderd: 17
Verwijderd: 04

Fill out details on each readmission because of HG*

**If more than 1 readmission, please print/copy this page another time to fill out details on subsequent readmission(s)*

**Readmission No** |__|__| *(0-10)*			
**Weight at readmission*†** |__|__|__| *kg (35-250)*<br>**Unknown: -1*<br>*†First measured weight at/during readmission*			
**Urinary ketones***<br>**First measurement on day of readmission or during readmission*	O Negative	O Positive<br>| O +<br>O ++<br>O +++<br>O ++++<br>O Unknown	O Unknown
**Intravenous drip**	O No	O Yes<br>| **i.v. drip solution contained***<br>☐ NaCl<br>☐ Glucose<br>☐ KCl<br>☐ Other; ____________<br>**If in medical or nursing record only: 'i.v. drip according to protocol', fill out what is prescribed according to local protocol for HG*	O Unknown
**Was i.v. drip placed on day of readmission**	O No<br>| **Date of placement*** |__|__|-|__|__|-|__|__| *(dd-mm-yy)*<br>**Unknown: 99-99-99*	O Yes	O Unknown
**Was i.v. drip removed on day of discharge**	O No<br>| **Date of removal*** |__|__|-|__|__|-|__|__| *(dd-mm-yy)*<br>**Unknown: 99-99-99*	O Yes	O Unknown
**Tube feeding**	O No	O Yes	O Unknown
**Was tube placed on day of readmission**	O No<br>| **Date of placement*** |__|__|-|__|__|-|__|__| *(dd-mm-yy)*<br>**Unknown: 99-99-99*	O Yes	O Unknown
**Was tube removed on day of discharge**	O No<br>| **Date of removal*†** |__|__|-|__|__|-|__|__| *(dd-mm-yy)*<br>**Unknown: 99-99-99*<br>*†Patients are normally discharged with tube in situ. Date may be later than date of discharge*	O Yes	O Unknown
**Antiemetics**	O No	O Yes<br>| ☐ Emesafene p.o. / supp<br>☐ Primperan p.o. / supp<br>☐ Primperan i.v.<br>☐ Ondansetron(Zofran) p.o. / supp<br>☐ Ondansetron (Zofran) i.v.<br>☐ Steroids*<br>☐ Other<br>**Prescribed as antiemetic, not because of fetal lung ripening*	O Unknown
**Vitamins**	O No	O Yes<br>| ☐ B1 *(thiamin)*<br>☐ B6 *(pyridoxin)*<br>☐ B12 *(cobalamin)*<br>☐ B complex<br>☐ Other	O Unknown

Verwijderd: 1

Verwijderd: 17

Verwijderd: 04

8. Delivery

Onset of labour
O Spontaneously
O Primary caesarean section
O Induction

Induction of labour or primary caesarean section
O No O Yes O Unknown
|
☐ Maternal indication*
☐ Fetal indication†
☐ Elective§

Such as hypertension
†Such as growth restriction or breech position
§Non-medical reason such as patients wish or 'DDD' (discrepantie draagkracht draaglast)

Place of birth
O Home delivery, primary care
O Hospital delivery, primary care
O Hospital delivery, secondary/tertiary care

Analgesics during labour*
O No O Yes O Unknown

**Not* during surgical intervention, e.g. caesarean section*

Route of delivery
O vaginally
O caesarean section

Delivery of placenta
O Spontaneously
O Manual removal
O Manual removal during caesarean section

Placental weight measured
O No O Yes O Unknown
|
Placental weight* |__|__|__|__| *grams (100-2000)*
**Unknown: -1*

Verwijderd: 1
Verwijderd: 17
Verwijderd: 04

Total haemorrhage |__|__|__|__| *ml (50-9999)*

Maternal death*†
O No
O Yes
|
Date of death |__|__| - |__|__| - |__|__| *(dd-mm-yy)*

**Within 6 weeks of delivery*
†Fill out SAE form

Verwijderd: 1
Verwijderd: 17
Verwijderd: 04

9. Neonatal birth data

Multiple pregnancy
O No O Yes
|
Child no. * |__| *(0-3)*

**If multiple, please print/copy this page another time to fill out details for subsequent child(ren)*

Date of birth |__|__| - |__|__| - |__|__| *(dd-mm-yy)*

Live birth
O Yes
O No, deceased during delivery < 24 hours postpartum*
O No, deceased before delivery*
|
Estimated date of death |__|__| - |__|__| - |__|__| *(dd-mm-yy)*

**Fill out SAE form*

Apgar score (5 min) |__|__| *(00-10)*

Umbilical cord pH's measured
O No O Yes O Unknown
|
Arterial pH |__|.|__|__| *(6.00-7.70)*
Venous pH |__|.|__|__| *(6.00-7.70)*

Sex
O Boy
O Girl

Birth weight |__|__|__|__| *grams (300-6500)*

Neonatal death ≥ 24 hours postpartum
O No O Yes* O Unknown
|
Date of death |__|__| - |__|__| - |__|__| *(dd-mm-yy)*

**Fill out SAE form*

Verwijderd: 1
Verwijderd: 17
Verwijderd: 04

10. Hospital admission postpartum

10.1 Maternal hospital admission

Maternal hospital admission < 12 hours postpartum

O No O Yes* O Unknown

|

☐ Maternal indication

☐ Neonatal indication

**Fill out SAE form*

If hospital admission on maternal indication: indication(s) for admission

☐ Suspected infection

☐ Tromboembolic complication

☐ Hypertensive disorder

☐ Postpartum haemorrhage

☐ Post-caesarean

☐ Eclampsia/HELLP

☐ Other

Date of hospital admission equal to date of delivery

O No O Yes O Unknown

|

Date of hospital admission |__|__| - |__|__| - |__|__| *(dd-mm-yy)*

Date of discharge home |__|__| - |__|__| - |__|__| *(dd-mm-yy)*

Verwijderd: 1

Verwijderd: 17

Verwijderd: 04

10.2 Neonatal hospital admission

Multiple pregnancy
O No O Yes
|
Child no. * |__| *(0-3)*

**If multiple, please print/copy this page another time to fill out details for subsequent child(ren)*

Neonatal hospital admission <6 weeks postpartum
O No O Yes* O Unknown
|
☐ Maternal indication
☐ Neonatal indication

**Fill out SAE form*

If hospital admission on neonatal indication: indication(s) for admission*
☐ Small for gestational age (defined as birth weight < P10)
☐ Large for gestational age (defined as birth weight >P 90)
☐ Congenital anomaly or suspicion for abnormality
☐ Hypoglycaemia (i.v. treatment needed)
☐ Hyperbilirubinemia (phototherapy or transfusion needed)
☐ Infection/ Sepsis (suspected or proven positive culture)
☐ Convulsions
☐ Other

*As reported in the final discharge letter of pediatrician

Date of hospital admission equal to date of birth
O No O Yes O Unknown
|
Date of hospital admission |__|__| - |__|__| - |__|__| *(dd-mm-yy)*

Date of discharge home |__|__| - |__|__| - |__|__| *(dd-mm-yy)*

Note: please attach an anonymized copy of the final pediatric discharge letter; mark this letter with maternal case number on every page

Verwijderd: 1
Verwijderd: 17
Verwijderd: 04

11. (Serious) Adverse Events

In this pregnancy, has there been an adverse event(s) (AE)*

O No O Yes O Unknown

|

Please specify; ___________________________

**Any undesirable event during the course of the study whether or not considered related to the intervention, spontaneously reported by patient, or observed by caregiver/research staff, but not leading to hospital admission (see SAE). Examples of AE's are: broken leg, fall from stairs, brain contusion etc.*

In this pregnancy, has there been a Serious Adverse Event(s) (SAE)*

O No O Yes O Unknown

|

☐ Miscarriage
☐ Hospital admission(s) after initial admission at study entry†
☐ Significant prolongation of hospital admission
☐ Maternal (pregnancy) complications§
☐ Complications due to tube placement
(e.g. aspiration/intestinal bleeding/intestinal perforation)
☐ Maternal death
☐ Neonatal hospital admission¥
☐ Birth defect/congenital anomaly neonate
☐ Perinatal death
☐ Other; _______________________________

**Any of the above mentioned situations, or AE causing significant disability to the patient, or AE requiring interventions to prevent a SAE from happening.*
†Any reason, excluding hospital admissions for HG (these are already reported in CRF)
§Think of ICU admission (e.g. due to massive postpartum haemorrhage), uterine rupture, placental abruption etc.
¥Any reason

Note: in all cases of SAE fill out SAE form (see study website → documents)

Verwijderd: 1
Verwijderd: 17
Verwijderd: 04

12. End Study Form

I have filled out this form accurately and truthfully

Name: ________________________________

Date: |__|__| - |__|__| - |__|__| *(dd-mm-yy)*

Signature: _____________________________

12.1 Study stop

Has patient reached the endpoint of the study (childbirth)
O No O Yes O Lost to follow up

Has patient (partially) withdrawn from study
O No O Yes
|
☐ Withdrawn from biobank
☐ Withdrawn from filling out diaries
☐ Completely withdrawn from study participation*

**Patient has explicitly expressed wish that patient information may no longer be used for this study*

Reason for (partial) study withdrawal
O Unknown
O Adverse event
O Other; ________________

END OF Case Report Form
THANK YOU FOR FILLING OUT THIS FORM

Verwijderd: 1
Verwijderd: 17
Verwijderd: 04

13. Supplement for patients included before 01-10-2014

Note: Is the patient included before 01-10-2014? Please try to answer the following questions.
For patients included from 01-10-2014 onwards, the following questions have been asked in questionnaire A (at inclusion) and filling out this supplement is not necessary

13.1 Maternal background

Ethnicity
O Unknown
O Dutch
O European / North American / Oceanean
O South American
O Turkish
O Surinamese
O Antillean / Aruban / Cape Verdian
O African (Sub-Sahara)
O Indonesian / Moluccan / Japanese
O Indian / Pakistani
O Other; ________________________

Highest finished education
O Unknown
O Primary school
O Secondary school
O Lower professional school *(VMBO)*
O Medium professional school *(MBO)*
O Higher professional school *(HBO)*
O University (WO)
O Other; ________________________

Relationship
O Unknown
O Single
O Living together with partner

Verwijderd: 1
Verwijderd: 17
Verwijderd: 04

13.2 Current pregnancy

Mode of conception
O Unknown
O Spontaneous (natural route)
O IUI (intrauterine insemination)
O Ovulation-induction (medication to effectuate menstrual cycle)
O IVF (in vitro fertilization)
O ICSI (intracytoplasmatic sperm injection)
O Other; ________________________

Nausea reported since gestational age |__|__| weeks *(0-20)*

Vomiting reported since gestational age |__|__| weeks *(0-20)*

Verwijderd: 1
Verwijderd: 17
Verwijderd: 04
